# Presynaptic Glycine Receptors Increase GABAergic Neurotransmission in Rat Periaqueductal Gray Neurons

**DOI:** 10.1155/2013/954302

**Published:** 2013-09-01

**Authors:** Kwi-Hyung Choi, Michiko Nakamura, Il-Sung Jang

**Affiliations:** Department of Pharmacology, School of Dentistry, Kyungpook National University, Daegu 700-412, Republic of Korea

## Abstract

The periaqueductal gray (PAG) is involved in the central regulation of nociceptive transmission by affecting the descending inhibitory pathway. In the present study, we have addressed the functional role of presynaptic glycine receptors in spontaneous glutamatergic transmission. Spontaneous EPSCs (sEPSCs) were recorded in mechanically dissociated rat PAG neurons using a conventional whole-cell patch recording technique under voltage-clamp conditions. The application of glycine (100 *µ*M) significantly increased the frequency of sEPSCs, without affecting the amplitude of sEPSCs. The glycine-induced increase in sEPSC frequency was blocked by 1 *µ*M strychnine, a specific glycine receptor antagonist. The results suggest that glycine acts on presynaptic glycine receptors to increase the probability of glutamate release from excitatory nerve terminals. The glycine-induced increase in sEPSC frequency completely disappeared either in the presence of tetrodotoxin or Cd^2+^, voltage-gated Na^+^, or Ca^2+^ channel blockers, suggesting that the activation of presynaptic glycine receptors might depolarize excitatory nerve terminals. The present results suggest that presynaptic glycine receptors can regulate the excitability of PAG neurons by enhancing glutamatergic transmission and therefore play an important role in the regulation of various physiological functions mediated by the PAG.

## 1. Introduction

Glycine, in addition to GABA, is the primary inhibitory neurotransmitter in the brain stem and spinal cord. In mature neurons, the inhibitory action of glycine is accomplished by activating strychnine-sensitive glycine receptors and opening Cl^−^ channels, which results in membrane shunting or hyperpolarization of postsynaptic neurons [1]. Glycine receptors are found in most of brain areas including the hippocampus, amygdala, ventral tegmental area, and periaqueductal gray (PAG) [2–5]. Nevertheless, functional roles of glycine receptors are largely unknown because glycine is unlikely to be released from presynaptic nerve terminals, and there is no direct evidence for glycinergic inhibitory postsynaptic currents in these brain structures (but see also [6]). However, previous studies have shown that endogenous glycine and/or taurine can elicit the tonic Cl^−^ currents mediated by glycine receptors in central neurons [5, 7], suggesting that endogenous glycine and/or taurine may play a role in the regulation of neuronal excitability. On the other hand, glycine receptors are also found in presynaptic nerve terminals of many brain regions, and their activation is known to facilitate neurotransmitter release from presynaptic nerve terminals [8–11]. In these cases, presynaptic glycine receptors might regulate the neuronal excitability in an indirect manner via the presynaptic modulation of neurotransmitter release.

The PAG is involved in the various functions including pain, vocalization, fear and anxiety, lordosis, and cardiovascular control [12, 13]. In particular, the PAG plays a crucial role in the regulation of nociceptive transmission as the PAG is one of regulatory centers affecting the endogenous descending inhibitory pathway such as noradrenergic and serotonergic systems [13]. In fact, electrical stimulation of the PAG region reduces neuropathic pain by activating the descending inhibitory system [14, 15]. In addition, the PAG is known to be one of the target sites for opioids and cannabinoids [16, 17]. On the other hand, it has been well established that several neurotransmitters including glutamate and GABA within the PAG are responsible for the regulation of nociceptive transmission [13, 18]. Of them, glycine is likely to play a role in the processing of pain within the PAG, as glycine is inversely correlated to nociceptive paw stimulation [19]. However, a significant amount of the glycine released within the PAG seems to act on glycine sites of NMDA receptors rather than strychnine-sensitive glycine receptors [20–22], indicating that the functional roles of glycine receptors in the PAG are still largely unknown. In the present study, therefore, we have investigated whether functional glycine receptors exist on glutamatergic nerve terminals projecting to PAG neurons and whether their activation modulates spontaneous glutamatergic transmission.

## 2. Materials and Methods

### 2.1. Preparation

All experiments complied with the guiding principles for the care and use of animals approved by the Council of the Physiological Society of Korea and the National Institutes of Health Guide for the Care and Use of Laboratory Animals, and every effort was made to minimize both the number of animals used and their suffering.

Sprague Dawley rats (12–16 d old, either sex) were decapitated under ketamine anesthesia (100 mg/kg, i.p.). The midbrain was dissected and transversely sliced at a thickness of 400 *μ*m using a microslicer (VT1000S; Leica, Nussloch, Germany). The midbrain slices containing the PAG were kept in an incubation solution (in mM: 124NaCl, 3KCl, 1.5KH_2_PO_4_, 24NaHCO_3_, 2CaCl_2_, 1.3MgSO_4_, and 10 glucose) saturated with 95% O_2_ and 5% CO_2_ at room temperature (22–24°C) for at least 1 h before the mechanical dissociation. For dissociation, slices were transferred into a 35 mm culture dish (Primaria 3801; Becton Dickinson, Rutherford, NJ, USA) containing a standard external solution (in mM: 150NaCl, 3KCl, 2CaCl_2_, 1MgCl_2_, 10 glucose, 10 Hepes, and pH 7.4 with Tris-base), and the PAG region was identified under a binocular microscope (SMZ-1; Nikon, Tokyo, Japan). Details of the mechanical dissociation have been described previously [23, 24]. Briefly, mechanical dissociation was accomplished using a custom-built vibration device and a fire-polished glass pipette oscillating at about 50–60 Hz (0.3–0.5 mm) on the surface of the ventrolateral PAG region. Slices were removed and the mechanically dissociated neurons were left for 15 min to allow the neurons to adhere to the bottom of the culture dish.

### 2.2. Electrophysiology

All electrophysiological measurements were performed using conventional whole-cell patch recording mode at holding potentials (*V*_H_ values) of −60 to −65 mV, which are the reversal potential of glycine-induced membrane currents determined in every PAG neurons, except where indicated (Axopatch 200B; Molecular Devices, Union City, CA, USA). Patch pipettes were made from borosilicate capillary glass (1.5 mm outer diameter, 0.9 mm inner diameter; G-1.5; Narishige, Tokyo, Japan) by use of a pipette puller (P-97; Sutter Instrument Co., Novato, CA, USA). The tip of pipette was firstly filled with the Cs-methanesulfonate-based internal solution (in mM: 140 Cs-methanesulfonate, 10CsCl, 2 EGTA, 5 QX-314, 2 ATP-Mg, 10 Hepes, and pH 7.2 with Tris-base) using a capillary phenomenon, and then the CsF-based internal solution, in which Cs-methanesulfonate was replaced with equimolar CsF, was backfilled using a syringe. The resistance of the recording pipettes filled with these internal solutions was 4–6 MΩ. The liquid junction potential (~−11 mV, measured by exchanging bath solution from internal solution to standard external solution) and pipette capacitance were compensated for. Neurons were viewed under phase contrast on an inverted microscope (TE2000; Nikon). Membrane currents were filtered at 2 kHz, digitized at 5 kHz, and stored on a computer equipped with pCLAMP 10.2 (Molecular Devices). During the recordings, 10 mV hyperpolarizing step pulses (30 ms in duration) were periodically applied to monitor the access resistance. All experiments were performed at room temperature (22–25°C).

### 2.3. Data Analysis

Spontaneous excitatory postsynaptic currents (sEPSCs) were counted and analyzed using the MiniAnalysis program (Synaptosoft, Inc., Decatur, GA, USA) as described previously [25]. Briefly, sEPSCs were screened automatically using an amplitude threshold of 10 pA and then were visually accepted or rejected based upon the rise and decay times. Basal noise levels during voltage-clamp recordings were typically less than 8 pA. The average values of the frequency, amplitude, and decay time constant (90–37%) of sEPSCs during the control period or each drug condition (5 min) were calculated for each recording, and the frequency and amplitude of all the events during the glycine application (1-2 min) were normalized to these values. The effects of these different conditions were quantified as a percentage increase in sEPSC frequency compared to the control values. The interevent intervals and amplitudes of a large number of synaptic events obtained from the same neuron were examined by constructing cumulative probability distributions and compared using the Kolmogorov-Smirnov (K-S) test with Stat View software (SAS Institute, Inc., Cary, NC, USA). Numerical values are expressed as the mean ± standard error of the mean (SEM) using values normalized to the control. Significant differences in the mean amplitude and frequency were tested using Student's paired two-tailed *t*-test, using absolute values rather than normalized ones. Values of *P* < 0.05 were considered significant.

### 2.4. Drugs

The drugs used in the present study were glycine, strychnine, 6-imino-3-(4-methoxyphenyl)-1(6H)-pyridazinebutanoic acid HBr (SR95531), tetrodotoxin (TTX), 6-cyano-7-nitroquinoxaline-2,3-dione (CNQX), DL-2-amino-5-phosphonovaleric acid (APV), QX-314, EGTA, CdCl_2_, and ATP-Mg (from Sigma, St. Louis, MO, USA). The standard external solution routinely contained 10 *μ*M SR95531 and APV 50 *μ*M APV to block GABA_A_ and NMDA receptors, respectively. All solutions containing drugs were applied using the “Y-tube system” for rapid solution exchange [26].

## 3. Results

**Figure 1:**
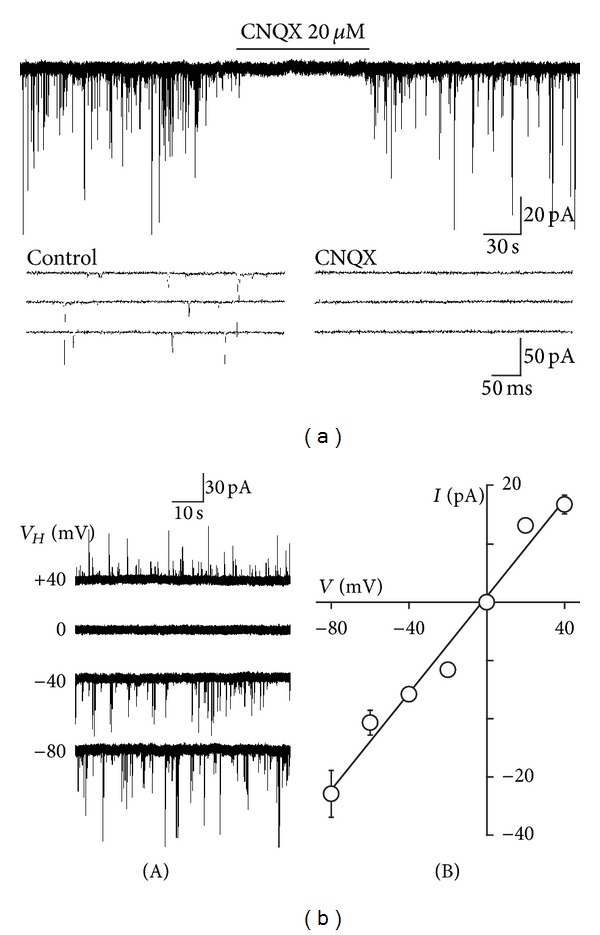
Glutamatergic sEPSCs recorded from acutely isolated PAG neurons. (a) A typical trace of glutamatergic sEPSCs observed before, during, and after application of 20 *μ*M CNQX, an AMPA/KA receptor blocker, at a *V*_H_ of 0 mV in the presence of 10 *μ*M SR95531 and 50 *μ*M APV, selective GABA_A_, and NMDA receptor antagonists, respectively. Insets represent typical traces with an expanded time scale. (b) (A) Typical traces of glutamatergic sEPSCs at various holding potentials (*V*_H_). (B) A plot of the mean amplitude of sEPSCs at various *V*_H_ values. The reversal potential was −2.5 mV, which is close to the theoretical equilibrium potential of monovalent cations. Each point was the mean and SEM from the 4 neurons.

After brief mechanical dissociation of the ventrolateral PAG region, several kinds of neurons that differed in soma diameter (10–15 *μ*m) and shape (multipolar, bipolar, and pyramidal-shaped) were found. These morphological properties of acutely isolated neurons were similar to those of PAG neurons identified in previous studies [27, 28]. When these neurons were held at a *V*_H_ of −60 mV using the whole-cell patch-clamp technique, the spontaneous inward synaptic currents were recorded in the presence of both 10 *μ*M SR95531 and 50 *μ*M APV, selective GABA_A_ and NMDA, and receptor antagonists, respectively. These spontaneous inward currents were completely and reversibly blocked by 20 *μ*M CNQX (*n* = 5), an AMPA/KA receptor blocker (Figure 1(a)).  Figure 1(b) shows typical raw traces recorded at various *V*_H_ conditions and the current-voltage relationship (*n* = 4). The reversal potential for the spontaneous synaptic currents was estimated from the current-voltage relationship to be −2.5 mV. This value is very similar to the theoretical equilibrium potential of monovalent cations. These results indicate that the spontaneous synaptic events recorded from acutely isolated PAG neurons were AMPA/KA receptor-mediated sEPSCs.

**Figure 2:**
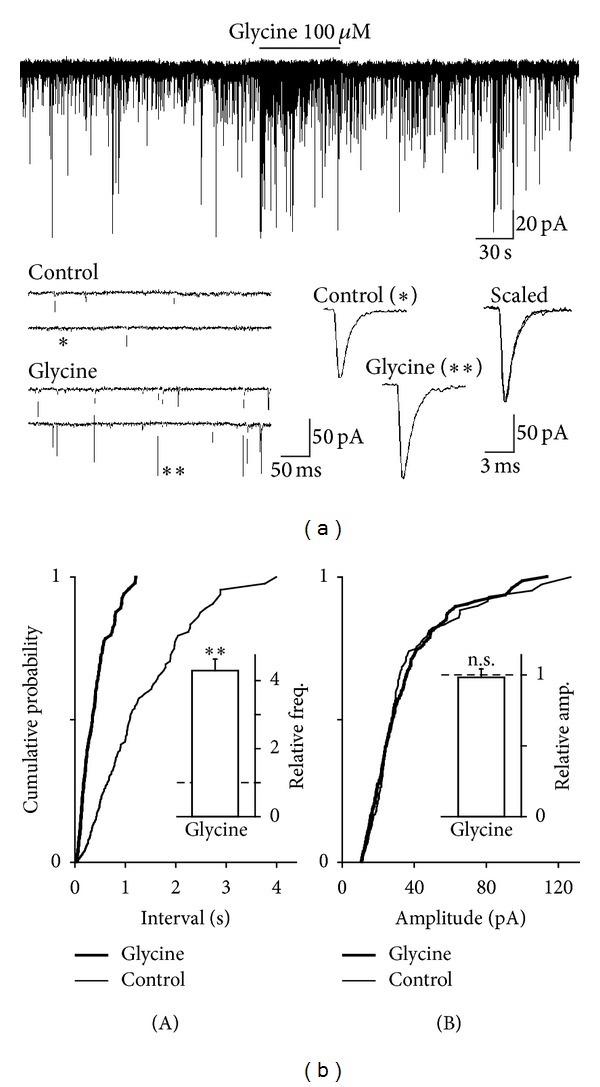
Effects of glycine on glutamatergic sEPSCs. (a) A typical trace of glutamatergic sEPSCs observed before, during, and after application of 100 *μ*M glycine. Insets represent typical traces with an expanded time scale (left) and single sEPSCs indicated by symbols (right). (b) Cumulative probability distribution for interevent interval (A) and current amplitude (B) of glutamatergic sEPSCs. 191 for control (thin lines) and 292 events for glycine (thick lines) were plotted. Insets column represents mean and SEM from 12 neurons. Dotted lines represent the relative control of basal frequency and amplitude of sEPSCs. **P < 0.01; n.s.: not significant.

To investigate whether excitatory nerve terminals projecting to PAG neurons express functional glycine receptors and whether the activation of presynaptic glycine receptors directly modulates spontaneous glutamate release, we observed the effect of exogenously applied glycine on sEPSCs. The glycine receptor-mediated membrane currents were minimized by using the CsF-based pipette solution and by adjusting the *V*_H_ to experimentally determined reversal potential of glycine-induced currents. In these conditions, glycine (100 *μ*M) rapidly and reversibly increased the frequency of glutamatergic sEPSCs (Figure 2(a)). In 12 neurons for which the effect was fully analyzed, glycine (100 *μ*M) increased sEPSC frequency to 429.7 ± 33.9% of the control (0.81 ± 0.18 Hz for control and 3.48 ± 0.27 Hz for glycine, *P* < 0.01), without affecting sEPSC amplitude (98.3 ± 5.9% of the control, 23.1 ± 1.9 pA for control, and 22.7 ± 1.6 pA for glycine, *P* = 0.57; Figures 2(a) and 2(b) insets). In addition, glycine significantly shifted the cumulative distribution of interevent interval to the left (*P* < 0.01, K-S test, Figure 2(b)(A)) without affecting the cumulative distribution of the current amplitude (*P* = 0.13, K-S test, Figure 2(b)(B)), consistent with an increase in the frequency of glutamatergic sEPSCs. Glycine also did not affect the decay time constant of glutamatergic sEPSCs (2.21 ± 0.12 ms of the control and 2.19 ± 0.13 ms for glycine, *P* = 0.96; Figure 2(a) inset). Taken together, these results suggest that glycine acts presynaptically to increase spontaneous glutamate release onto acutely isolated PAG neurons.

**Figure 3:**
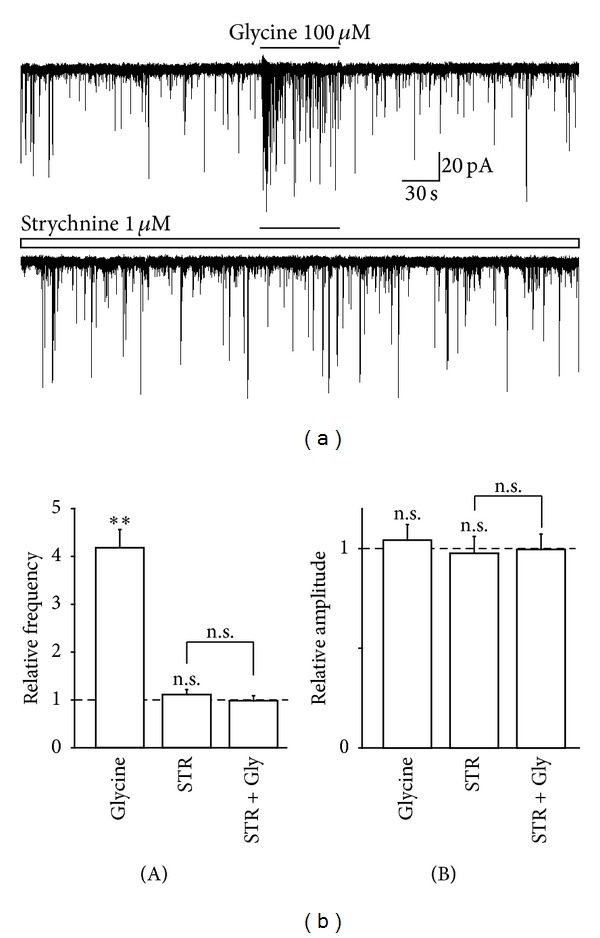
Effect of strychnine on glycine-induced increase in sEPSC frequency. (a) Typical traces of glutamatergic sEPSCs observed during the application of 100 *μ*M glycine in the absence (upper) and presence (lower) of 1 *μ*M strychnine. (b) Glycine-induced changes in frequency (A) and amplitude (B) of sEPSC in the absence and presence of strychnine. Each column was the mean and SEM from 6 neurons. **P < 0.01; n.s.: not significant.

To investigate whether the glycine-induced increase in spontaneous glutamate release is mediated by presynaptic glycine receptors, we observed the effect of strychnine, a specific glycine receptor antagonist, on the glycine-induced increase in sEPSC frequency. Strychnine (1 *μ*M) by itself had no effect on the basal frequency (111.6 ± 10.3% of the control, *n* = 6, *P* = 0.31) or amplitude (97.6 ± 8.5% of the control, *n* = 6, *P* = 0.21) of glutamatergic sEPSCs (Figures 3(a) and 3(b)). In the presence of 1 *μ*M strychnine, the facilitatory action of glycine (418.1 ± 38.3% of the control, *n* = 6, *P* < 0.01) was completely attenuated to 88.4 ± 9.2% of the strychnine condition (*n* = 6, *P* = 0.42, Figures 3(a) and 3(b)(A)).

**Figure 4:**
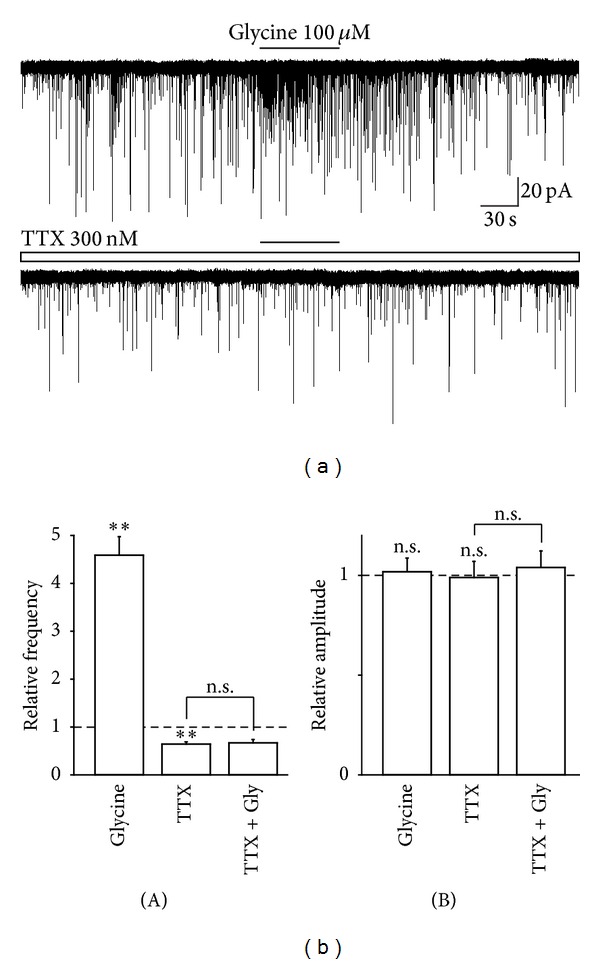
Effect of TTX on glycine-induced increase in sEPSC frequency. (a) Typical traces of glutamatergic sEPSCs observed during the application of 100 *μ*M glycine in the absence (upper) and presence (lower) of 300 nM TTX. (b) Glycine-induced changes in frequency (A) and amplitude (B) of sEPSC in the absence and presence of TTX. Note that the glycine-induced facilitation of sEPSC frequency was completely suppressed by TTX. Each column was the mean and SEM from 7 neurons. **P < 0.01; n.s.: not significant.

Next, the possible mechanisms underlying the glycine-induced increase in spontaneous glutamate release were examined. Since the activation of presynaptic glycine receptors facilitates spontaneous neurotransmitter release by eliciting a presynaptic depolarization [9–11], we observed the effect of TTX, a voltage-dependent Na^+^ channel blocker, on the glycine-induced increase in sEPSCs frequency. The application of 300 nM TTX significantly decreased the basal sEPSC frequency (64.4 ± 4.6% of the control, *n* = 6, *P* < 0.01, Figures 4(a) and 4(b)(A)), but it had no effect on the basal sEPSC amplitude (98.9 ± 8.1% of the control, *n* = 6, *P* = 0.17, Figures 4(a) and 4(b)(B)). In the presence of 300 nM TTX, the facilitatory action of glycine (458.6 ± 39.1% of the control, *n* = 6, *P* < 0.01) was completely occluded to 103.7 ± 11.0% of the TTX condition (*n* = 6, *P* = 0.55, Figures 4(a) and 4(b)(A)).

**Figure 5:**
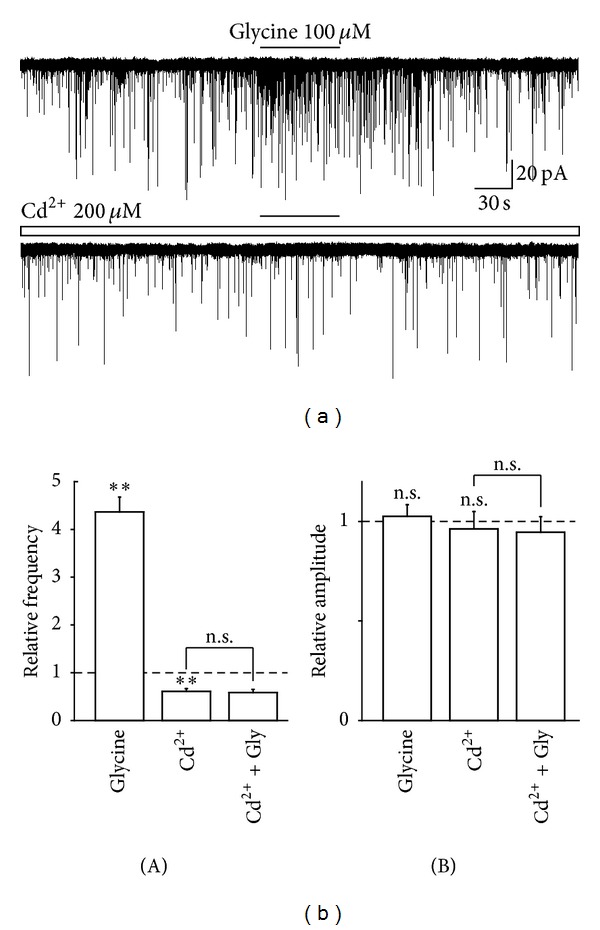
Effect of Cd^2+^ on glycine-induced increase in sEPSC frequency. (a) Typical traces of glutamatergic sEPSCs observed during the application of 100 *μ*M glycine in the absence (upper) and presence (lower) of 200 *μ*M Cd^2+^. (b) Glycine-induced changes in frequency (A) and amplitude (B) of sEPSC in the absence and presence of Cd^2+^. Note that the glycine-induced facilitation of sEPSC frequency was completely suppressed by Cd^2+^. Each column was the mean and SEM from 7 neurons. **P < 0.01; n.s.: not significant.

The neurotransmitter release is triggered by an increase in the intraterminal Ca^2+^ concentration, which is generally accomplished by presynaptic voltage-dependent Ca^2+^ channels (VDCCs) [29]. Therefore, we further examined the effect of Cd^2+^, a general VDCC blocker, on the glycine-induced increase in sEPSCs frequency. The application of 200 *μ*M Cd^2+^ also significantly decreased the basal sEPSC frequency (61.1 ± 5.5% of the control, *n* = 6, *P* < 0.01, Figures 4(a) and 4(b)(A)). However, Cd^2+^ did not affect the basal sEPSC amplitude (96.2 ± 8.8% of the control, *n* = 6, *P* = 0.61, Figures 5(a) and 5(b)(B)). In the presence of 200 *μ*M Cd^2+^, the facilitatory action of glycine (436.8 ± 31.1% of the control, *n* = 6, *P* < 0.01) was completely occluded to 95.9 ± 10.6% of the Cd^2+^ condition (*n* = 6, *P* = 0.28, Figures 5(a) and 5(b)(B)).

## 4. Discussion

Previous studies have shown that glycine receptors are expressed on presynaptic nerve terminals at central synapses and that their activation modulates the presynaptic release of a variety of neurotransmitters, such as glutamate [8, 11], GABA [10], and glycine [9]. Several lines of evidence suggest that glycine receptors are also expressed on excitatory nerve terminals projecting to PAG neurons and that their activation enhances spontaneous glutamate release onto PAG neurons. First, glycine significantly increased the frequency of sEPSCs without affecting the current amplitude, consistent with a presynaptic locus of glycine action. Second, this facilitatory action of glycine on glutamatergic sEPSCs was completely blocked by strychnine. Although presynaptic GABA_A_ receptors also enhance spontaneous glutamate release at these synapses [30], the involvement of presynaptic GABA_A_ receptors should be negligible because the present study was performed after the blockade of GABA_A_ receptors with SR95531. In addition, since the extracellular solution contained APV, a specific NMDA receptor antagonist, the involvement of possible NMDA receptors should be also negligible. Third, the preparation used in this study would support a presynaptic locus of glycine action because dissociated neurons have presynaptic nerve terminals without their parent soma [24].

In the present study, we found that glycine failed to enhance sEPSC frequency in the presence of either TTX or Cd^2+^, suggesting that the glycine-induced increase in sEPSC frequency requires the activation of voltage-dependent Na^+^  and Ca^2+^channels. That is, the activation of presynaptic glycine receptors might depolarize excitatory nerve terminals, and that this presynaptic depolarization seems to activate voltage-dependent Na^+^ and Ca^2+^ channels subsequently. In addition, since glycine had no facilitatory effect on spontaneous glutamate release in the presence of TTX, the extent of glycine receptor-mediated presynaptic depolarization might be not enough to activate VDCCs directly [25, 31]. Alternatively, glycine receptors might be expressed on preterminal region so that the glycine receptor-mediated depolarization would affect voltage-dependent Na^+^ rather than Ca^2+^ channels at axons. Similarly, nicotinic acetylcholine receptors expressed on the axonal region are known to enhance spontaneous neurotransmitter release in a TTX-sensitive manner [32]. On the other hand, given that glycine receptors are permeable to Cl^−^  but not cations and that the activation of glycine receptors elicits a presynaptic depolarization; excitatory nerve terminals projecting to PAG neurons might maintain higher intraterminal Cl^−^ concentration than that predicted for passive Cl^−^ distribution. This can be accomplished by the inwardly directed Cl^−^ cotransporters such as bumetanide-sensitive Na^+^-K^+^-2Cl^−^ cotransporter type 1 [33, 34]. Similarly, we have previously shown that bumetanide-sensitive Na^+^-K^+^-2Cl^−^ cotransporter type 1 maintains the higher Cl^−^ concentration within presynaptic nerve terminals [25, 35]. In this regard, since the Cl^−^ concentration within the neuronal soma becomes lower with postnatal development by changing the expression of Cl^−^ cotransporters [36], it is of interest to examine whether the expression of presynaptic Cl^−^ cotransporters as well as the glycine receptor-mediated presynaptic modulation alters during postnatal development. 

As PAG neurons project their excitatory axon terminals directly to serotonergic and noradrenergic neurons of the medulla, which innervate their fibers the superficial dorsal horn [37], the excitability of PAG neurons should be a key factor involved in the PAG-mediated descending inhibitory systems. For example, microinjection of the GABA_A_ receptor antagonists or glutamate into the PAG shows antinociceptive responses in animal models [38–40]. In addition, opioid analgesics seem to disinhibit tonically active GABAergic neurons within the PAG [41], suggesting that an increase in the excitability of output PAG neurons produces analgesia. In this regard, glycine might be also involved in the regulation of excitability of PAG neurons. For example, a previous study has shown that the microinjection of glycine into the dorsal PAG of rats increases tail-flick latencies in a dose-dependent manner, and this hyponociceptive effect of glycine is reversed by coadministration with the specific inhibitor for NMDA receptor glycine site [42], suggesting that microinjected glycine acts on glycine-binding site of NMDA receptors to elicit hyponociception. In addition, a recent study has shown that the microinjection of glycine into the ventrolateral PAG of rats produces conflicting results, for example, hyperalgesia or analgesia [43]. In this study, while the glycine-induced analgesia is blocked by the NMDA receptor antagonist, the glycine-induced hyperalgesia is blocked by the glycine receptor antagonist [43], suggesting that glycine acts as an excitatory transmitter, for example, coagonist for NMDA receptors, to increase the excitability of output PAG neurons. In the case of glycine-induced hyperalgesia, the activation of glycine receptors, presumably somatodendritic and/or postsynaptic glycine receptors, might result from the decrease in the excitability of output PAG neurons. Although the source of extracellular glycine remains to be elucidated, glycine might be synaptically released as described previously [44]. It should be noted that, however, postsynaptic glycine receptors are unlikely to contribute to the regulation of neuronal excitability, as inhibitory postsynaptic currents are absolutely mediated by GABA_A_ receptors rather than strychnine-sensitive glycine receptors [45]. 

In the present study, we have shown that the activation of presynaptic glycine receptors increases spontaneous glutamate release onto PAG neurons via a presynaptic depolarization. The present results would provide a physiological role of presynaptic glycine receptors in the antinociceptive function mediated by the PAG, as the activation of presynaptic glycine receptors can increase the excitability of PAG neurons by enhancing excitatory glutamatergic transmission. This speculation might be different from previous findings showing that glycine microinjected into the PAG produces hyperalgesic action in a strychnine-sensitive manner [43]. However, the previous behavioral findings might be not applicable to the present study because the microinjected glycine can activate somatodendritic as well as presynaptic glycine receptors within the PAG region. Although it is still unknown whether the glycine-induced hyperalgesia is mediated by somatodendritic or presynaptic glycine receptors, somatodendritic glycine receptors might be responsible for the microinjected glycine-induced hyperalgesia. This is because the glycine-induced hyperpolarization decreases the excitability of output PAG neurons, as described above. In fact, PAG neurons express functional somatodendritic glycine receptors, and the application of glycine to isolated PAG neurons elicits large Cl^−^  currents [46]. Further electrophysiological and behavioral studies will be needed to elucidate the differential roles of somatodendritic and presynaptic glycine receptors in the regulation of nociceptive transmission mediated by the PAG.

## 5. Conclusions

In conclusion, we have shown that functional glycine receptors are expressed on glutamatergic nerve terminals projecting to PAG neurons and that the activation of presynaptic glycine receptors depolarizes presynaptic terminals to enhance spontaneous glutamate release. The present results suggest that presynaptic glycine receptors can regulate the excitability of PAG neurons by enhancing glutamatergic transmission and therefore play an important role in the regulation various physiological functions mediated by the PAG.
