# Correction to “Presynaptic Glycine Receptors Increase Glutamatergic Neurotransmission in Rat Periaqueductal Gray Neurons”

**DOI:** 10.1155/np/9785837

**Published:** 2026-02-09

K‐W. Choi, M. Nakamura, and I‐S. Jang, “Presynaptic Glycine Receptors Increase Glutamatergic Neurotransmission in Rat Periaqueductal Gray Neurons,” *Neural Plasticity*, 2013, 954302, https://doi.org/10.1155/2013/954302.

In the article, there was an error in the title, which has now been corrected from:

“Presynaptic Glycine Receptors Increase GABAergic Neurotransmission in Rat Periaqueductal Gray Neurons”

To:

“Presynaptic Glycine Receptors Increase Glutamatergic Neurotransmission in Rat Periaqueductal Gray Neurons”

We apologize for this error.

